# Potential for synergy in soil inoculation for nature restoration by mixing inocula from different successional stages

**DOI:** 10.1007/s11104-018-3825-0

**Published:** 2018-10-03

**Authors:** E. R. Jasper Wubs, Pauline D. Melchers, T. Martijn Bezemer

**Affiliations:** 1 Department of Terrestrial Ecology, Netherlands Institute of Ecology (NIOO-KNAW), P.O. Box 50, 6700 AB Wageningen, The Netherlands; 2 Laboratory of Nematology, Wageningen University and Research (WUR), P.O. Box 8123, 6700 ES Wageningen, The Netherlands; 3 Institute of Biology, Section Plant Ecology and Phytochemistry, Leiden University, PO Box 9505, 2300 RA Leiden, The Netherlands

**Keywords:** Antagonists, Community coalescence, Mutualists, Plant-soil interactions, Soil inoculation

## Abstract

**Background and aims:**

Soil inoculation is a powerful tool for the restoration of terrestrial ecosystems. However, the origin of the donor material may differentially influence early- and late-successional plant species. Donor soil from late-succession stages may benefit target plant species due to a higher abundance of soil-borne mutualists. Arable soils, on the other hand, may suppress ruderals as they support more root herbivores that preferentially attack ruderal plant species, while mid-succession soils may be intermediate in their effects on ruderals and target species performance. We hypothesized that a mixture of arable and late-succession inocula may outperform pure late-successional inocula for restoration, by promoting late-successional target plants, while simultaneously reducing ruderal species’ performance.

**Methods:**

We conducted a glasshouse experiment and tested the growth of ruderal and target plant species on pure and mixed inocula. The inocula were derived from arable fields, mid-succession grasslands and late-succession heathlands and we created a replacement series testing different pairwise mixitures for each of these inocula types (ratios: 100:0, 75:25, 50:50, 25:75, 0:100 of inoculum A and B respectively).

**Results:**

In general, we found that a higher proportion of heathland material led to a higher aboveground biomass of target plant species, while responses of ruderal species were variable. We found synergistic effects when specific inocula were mixed. In particular, a 50:50 mixture of heathland and arable soil in the inoculum led to a significant reduction in ruderal species biomass relative to the two respective pure inocula. The overall response was driven by *Myosotis arvensis*, since the other two ruderal species were not significantly affected.

**Conclusions:**

Mixing inocula from different successional stages can lead to synergistic effects on restoration, but this highly depends on the specific combination of inocula, the mixing ratio and plant species. This suggest that specific inocula may need to be developed in order to rapidly restore different plant communities.

**Electronic supplementary material:**

The online version of this article (10.1007/s11104-018-3825-0) contains supplementary material, which is available to authorized users.

## Introduction

Many natural ecosystems need to be restored in order to reach international conservation targets (Vitousek et al. 1997; Hobbs and Harris 2001). Plant and soil communities tightly interact and plant-soil interactions are thought to play a key role during ecological restoration (Reynolds et al. 2003; Eviner and Hawkes 2008; Kardol and Wardle 2010). Soil inoculation, where entire (late-successional) soil communities are translocated to areas to be restored can be a powerful tool to rapidly restore terrestrial ecosystems (Harris 2009; Wubs et al. 2016). Using a large-scale field experiment in an area intended for nature restoration on former arable land, we recently showed that application of whole soil inocula sourced from a target grassland or heathland can steer the above- and belowground community composition in the ecosystem under restoration in the direction of its respective donor (Wubs et al. 2016). How the composition of soil inocula can be optimized and whether combinations of different inoculum sources can lead to synergistic effects on the performance of ruderal and restoration target species is unclear.

The net effect of the soil community on plants is determined by the balance of the actions of plant antagonists, symbionts and decomposers (Van der Putten et al. 2016). Antagonists include soil-borne pathogens and root herbivores, while symbionts include mycorrhizal fungi and plant-growth promoting rhizobacteria. The composition, and abundance of the soil community changes considerably during natural succession (Kardol et al. 2005; Van der Wal et al. 2006; Bauer et al. 2015; Castle et al. 2016; Frouz et al. 2016). For example, the abundance of nematode and insect root herbivores is initially high on arable fields and then declines during secondary succession (Brown and Gange 1992; Korthals et al. 2001; Verschoor et al. 2001; Kardol et al. 2005; Rasmann et al. 2011). Tillage in arable cultivation reduces the abundance and diversity of many soil taxa (Tsiafouli et al. 2015), including mycorrhizal fungi (Helgason et al. 1998). Subsequently, during secondary succession on former arable land the abundance of mycorrhiza increases (Janos 1980; Johnson et al. 1991; Barni and Siniscalco 2000) and their composition changes (Johnson et al. 1991; Barni and Siniscalco 2000). These patterns in soil community development during secondary succession may be used to optimize soil inocula using the community coalescent approach (sensu Rillig et al. 2016), whereby soil communities from different origins are brought into contact to generate novel soil communities.

For the successful restoration of species-rich late-successional plant communities, early-successional ruderal species need to be suppressed and late-successional target species promoted. Early-successional plants tend to be sensitive to antagonists (Kardol et al. 2006), while late-successional species, typically the target species for conservation and restoration, are more dependent on soil-borne symbionts (Reynolds et al. 2003; Kardol et al. 2007, 2013; Middleton and Bever 2012). For instance, root herbivores are known to feed selectively on early-successional ruderal plant species due to their higher palatability and low investment in defense (Brown and Gange 1992; Fraser and Grime 1999). Late-successional plants respond most strongly to inoculation with mycorrhizal fungi (Middleton and Bever 2012), particularly when locally sourced inoculum is used (Middleton et al. 2015). In addition, it has been shown, both in the glasshouse and in the field, that inoculation with a late-successional soil community can differentially affect the performance of ruderal and target species and that late-successional soil inocula can promote restoration success (De Deyn et al. 2003; Kardol et al. 2006; Carbajo et al. 2011; Middleton and Bever 2012; Wubs et al. 2016). However, even though the growth of late-successional plants was improved, they did not gain dominance over the early-successional plants in these experiments. To maximize the effectiveness of soil inoculation, the inoculum should both suppress ruderals and promote late-successional species. We hypothesize this can be achieved by mixing inocula from both early- and late-successional origin. To our knowledge this has not been tested.

**Fig. 1:**
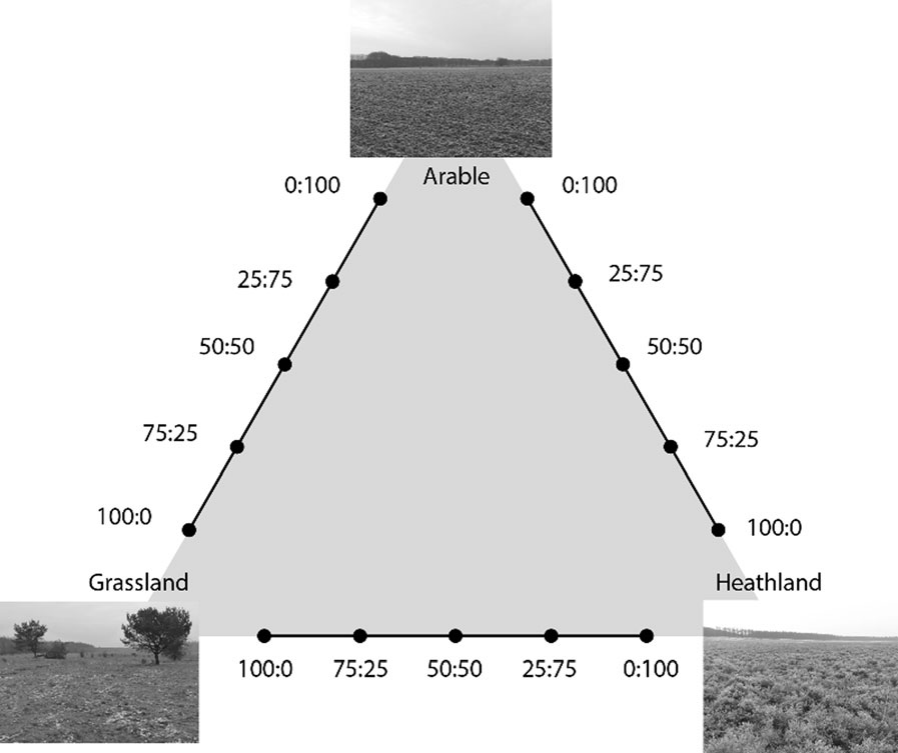
Experimental design. Inocula of arable fields, mid-succession grasslands, and dry heathlands were mixed in replacement series. The photos show one field of each type at the time of sampling. This design was replicated over three sets of fields (field triplets; Table S1), and four replicates per field triplet (i.e. per treatment *n* = 3 × 4 = 12)

In this study, we tested how different mixtures of soil inocula affect the performance of ruderal and target plant species (Fig. 1). We tested mixtures of soils sampled from arable fields, mid-succession grasslands and late-succession heathlands, by creating a replacement series among each pair of inoculum sources. The replacement series design was borrowed from plant competition experiments (De Wit 1960; Weigelt and Jolliffe 2003), and are characterized by a constant total amount of inoculum soil across treatments, but varying relative amounts of the two inoculum sources that are mixed (here ratios: 100:0, 75:25, 50:50, 25:75, 0:100 of inoculum A and B respectively). We expected that inocula with a high proportion of material from heathlands would promote the performance of the target plant species and that inocula with a high proportion of arable field soil would suppress ruderals. Mid-succession grassland soils were expected to have intermediate effects on target and ruderal species as they still contain relatively high amounts of soil-borne antagonists, but also mutualists. This could mean that 50:50 mixtures of arable field and heathland soil lead to the same effects as mid-succession grassland soil. However, positive and negative plant-soil feedback effects accrue and dissipate in complex ways over time as different plant re-condition the soil (Wubs and Bezemer 2018). Since the arable and heathland soil were more strongly conditioned by ruderals and late-succession target species respectively in previous years, we expect the effects of early-late inoculum mixtures to be more pronounced than the mid-succession grassland inoculum. Thus, we expected that a mixture of arable and heathland inoculum would lead to the best and worst performance of target and ruderal species respectively, because both their specific plant symbionts and antagonists would be present in the mixed inoculum. We explicitly tested for synergy among inoculum sources by comparing plant performance on mixed inocula to the expected performance based on the performance on pure inocula. A positive synergistic effect for the target plants, suggest that the mixed inoculum leads to higher biomass than might be expected based on the pure inocula it was derived from.

## Methods

We conducted a glasshouse experiment where a common background soil was inoculated with different soil inocula (9:1 w:w). Both the inocula and the background soil were collected in January 2015 on sandy or sandy loam glacial deposits in the central part of the Netherlands (Table S1). The background soil was collected from the Reijerscamp, an ex-arable field of 160 ha that has been undergoing restoration since 2006. We used the relatively nutrient rich organic top-soil (Table S1) of this site to 1) fit with eutrophic starting conditions in restoration projects in northwestern Europe and 2) even out differences in nutrient levels among the inocula by the ample nutrient availability in the background soil. The field has been in intensive agricultural use at least since World War 2 until 2004. Then, it was used for extensive wheat cultivation for two years prior to the implementation of large-scale restoration measures (Wubs et al. 2016). The soil was collected from the central part of the field, where the only management consisted of cattle grazing (25–30 cows throughout the year, roaming freely in the entire 160 ha field) and removal of tree seedlings (particularly *Betula* spp. and *Prunus serotina*). We collected soil from the organic layer within 10–50 cm depth (approximately 1300 kg), which was subsequently sieved over a 1 cm mesh to remove major roots and stones and homogenized. The common background soil was sterilized (>25KGray gamma radiation, Isotron, Ede, the Netherlands) to eliminate the resident soil community.

Three types of inocula were selected and sampled from three fields each within the study region (Table S1), that differ in soil community composition (Kardol et al. 2005; Van der Wal et al. 2006; Wubs et al. 2016; Morriën et al. 2017; Hannula et al. 2017). The types were: 1) arable fields (wheat or rye in recent years) in extensive organic cultivation with annual tillage (arable, A), 2) ex-arable grasslands that had undergone 27–33 yrs. of secondary succession (grassland, G), and 3) dry heathlands that have been in existence at least since the thirteenth century (heathland, H). We divided the nine fields into three groups of three (field triplet), each group containing one field of each type, based on geographic proximity. The distance between any pair of fields used for mixing inocula was between 0.7 and 5.7 km. Within each field an area of 5 × 5 m was selected at least 20 m from the edge of the field. At each corner of the selected area 5 kg of soil was collected from the upper 10–15 cm. The soil was sieved over a 1 cm mesh to remove stones and large roots. Upon return to the lab the four samples per field were pooled based on equal amounts of dry weight resulting in homogenized inoculum material of 20 kg per field. Three subsamples were taken per pooled inoculum and analyzed for chemical composition. The subsamples were oven-dried for five days at 40 °C and analyzed for soil organic matter content (24 h, 430 °C, loss on ignition), soil acidity (pH in 1:2.5 soil:H_2_O), and N (KCl-extraction) and P (Olsen’s extraction) content (Table S1). The ammonium, nitrate (both λ = 520) and phosphate (λ = 880 nm) concentration was measured colorimetrically using a QuAAtro Segmented Flow Analysis system (SEAL Analytical Netherlands, Rijen, The Netherlands).

Within each field triplet, inocula of each of the three types (arable, grassland and heathland) were mixed in a replacement series (Fig. 1). Mixtures were made for each pair of fields within a field triplet based on dry weight in five ratios: 100:0, 75:25, 50:50, 25:75, 0:100. For each replacement series, two separate sets of pure (i.e. 100% of one inoculum source) inocula replicates were included. Each treatment was replicated four times (3 triplets × 3 replacement series per triplet × 5 inocula mixing levels per series × 4 replicates = 180 experimental units).

The experiment was conducted in pots (17x17x17 cm) which were filled with soil containing 3.6 kg of sterilized background soil and 400 g inoculum (9:1 w:w dw) which was thoroughly mixed throughout the pot. We used six plant species as a test community, all of which are native to the study area. The species were selected based on their occurrence during secondary succession on sandy soils in the Netherlands (Schaminée et al. 1996, 1998) and seed availability at commercial growers. Three species were early-successional ruderals: *Crepis capillaris* (L.) Wallr. (Asteraceae), *Lolium perenne* L. (Poaceae) and *Myosotis arvensis* (L.) Hill (Boraginaceae), and three were late-successional, conservation target species: *Arnica montana* L. (Asteraceae), *Festuca filiformis* Pourr. (Poaceae) and *Campanula rotundifolia* L. (Campanulaceae), with one grass and two forbs in each group. Seeds were obtained from commercial suppliers of wild plant seeds (Cruydthoeck, Assen, the Netherlands and B&T World Seeds, Paguignan, France) and germinated (sterilized 1 min. in 5% NaClO solution) on moistened glass beads in a climate chamber (12 h light/dark cycle, 20 °C by day and 15 °C at night). Two individuals of each species were planted in a random position in a circle in the soil of each pot, so that all pots were planted with all six species. Any seedlings that died during the first two weeks were replaced. The pots were placed in the glasshouse in a randomized block design where the blocks corresponded to the field triplets (i.e. three blocks). The plants were allowed to grow in the glasshouse (16:8 h day: night, natural light supplemented with 600 W metal-halide lamps, 1 per 4 m^−2^, approx. 225 μmol light quanta m^−2^ s^−1^ at plant level, 21:16 °C day: night, 50–70% relative humidity) for 7 weeks and watered three times per week. Subsequently the shoots of each species were clipped at the soil surface, oven-dried for two days at 75 °C and weighed. Plant mortality was low during the experiment (1.3%).

### Data analysis

We analyzed univariate response data using linear mixed models (LMMs), with separate models for total biomass, ruderal biomass and target biomass and a separate model for each of the six plant species. We included a random effect for block in the analysis and a fixed effect for inoculum treatment. In addition, we tested how the plant species responded to each kind of inoculum material. To do so we conducted separate regression analyses, with the same random effects structure as above, for each replacement series (i.e. arable-grassland, arable-heathland, and grassland-heathland). Here the proportion of one of the two inoculum sources in the mixture was used as a continuous predictor (e.g. proportion arable, i.e. 0, 25, 50, 75 and 100, for arable-grassland mixture). We used a multiple response permutation procedure (MRPP) to test for overall differences in plant community composition, based on shoot biomass of each species in a pot. The pots were permuted within blocks only, to account for effects due to the sampling scheme (field triplets) and spatial positioning effects in the glasshouse (blocks).

We tested for synergy in the mixed inocula by calculating the expected performance of the ruderal and target plant species groups based on the pure inocula and the ratios in which their material was mixed. We then subtracted the expected performance from the actual observed performance in the pots with mixed inocula. The expected performance was calculated separately for each pair of fields. Differences between observed and expected performance were analyzed in LMMs with a random effect for blocks and mixed inoculum treatment (nine levels) as a fixed effect. Within these models significant deviations from zero (i.e. no synergistic effect) were tested as planned contrasts (Adbi and Williams 2010).

All analyses were conducted in R 3.3.1 (R Core Team 2017), LMMs were modelled using nlme 3.1–128 (Pinheiro et al. 2017), MRPP using vegan 2.4–1 (Oksanen et al. 2018). Model assumptions were checked graphically and heteroscedasticity was modelled using generalized least squares (Pinheiro and Bates 2000; Zuur et al. 2009). Post-hoc test were performed using the lsmeans 2.23–5 package (Lenth 2015) using the false discovery rate to correct for multiple comparisons (Benjamini and Hochberg 1995).

## Results

**Fig. 2:**
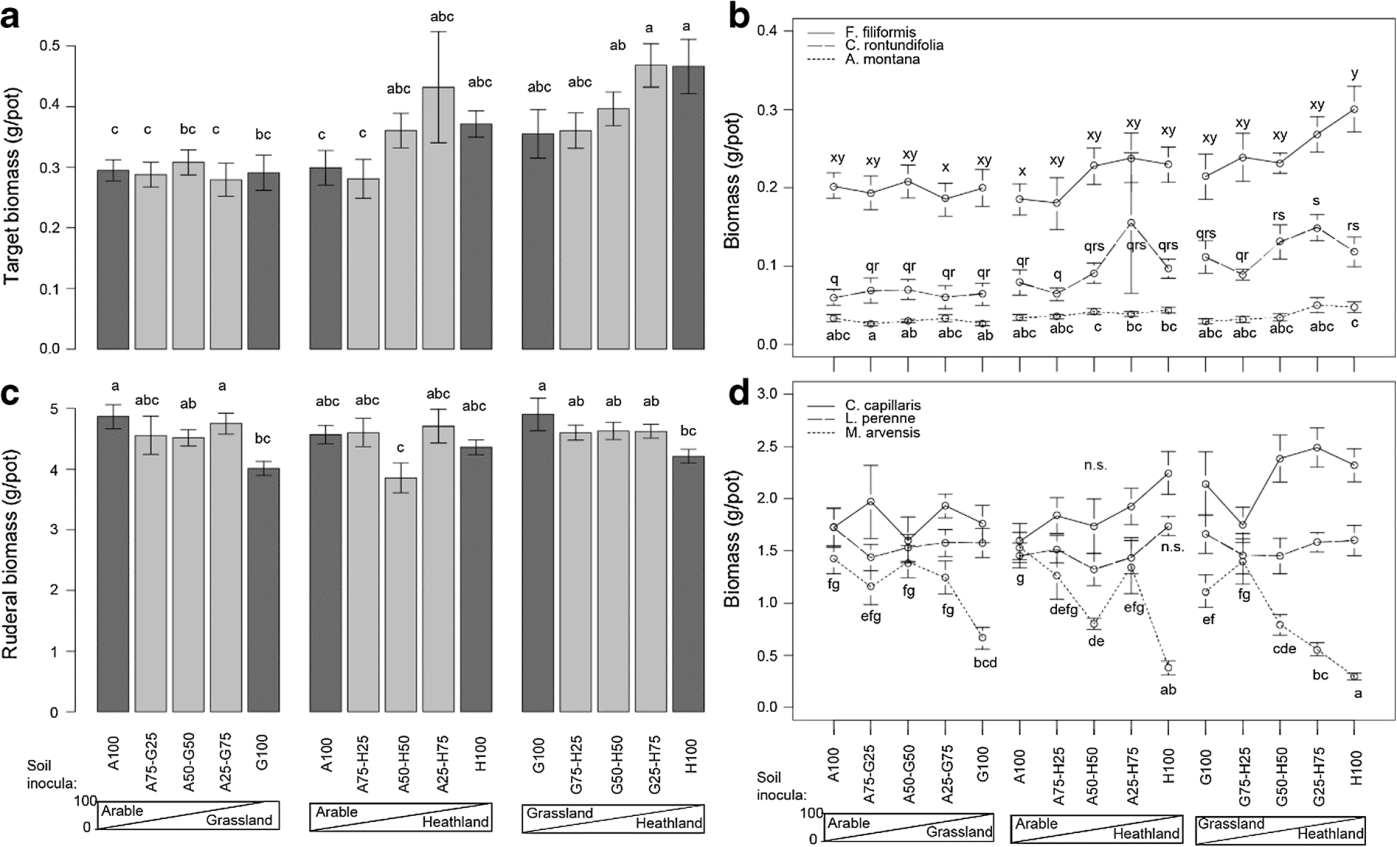
Target (**a**, **b**) and ruderal (**c**, **d**) species biomass (mean ± SE) per inoculum as species groups (**a**, **c**) and per species separately (**b**, **d**). Dark grey bars indicate the pure inocula (**a**, **c**), the different lines connect species within a replacement series (**b**, **d**). Different letters indicate significant differences among the treatments, see Table 1 for the overall analyses

In general, the composition of the soil inoculum influenced the plant community composition (Fig. 2; MRPP Pseudo-F = 4.07, *p* = 0.001). The biomass of the target species increased by 33% with an increasing fraction of heathland material in the inoculum (Fig. 2a; Table 1). Biomass of the ruderal species was lower in the A50-H50, G100 (A-G series) and H100 (G-H series) inoculum treatments compared to the other treatments (Fig. 2c; Table 1). The same pattern was found for total plant biomass (Fig. S1; Table 1). Surprisingly, the ruderal biomass in the two G100 treatments was significantly different among the two replacement series (A-G vs. G-H) in which they occurred (Fig. 2c).

**Table 1:** Results of the statistical analyses both as a one-way analysis with inoculum treatment as a fixed factor and as a regression analysis per replacement series. Values in bold are significant (*p* < 0.05). B = the direction of the relationship, e.g. H > A indicates that biomass is higher with a higher proportion of heathland material in the inoculum relative to arable soil. Relationships significant at p < 0.1 are shown in brackets

	1-way LMM			Regression analysis (per replacement series)											
	Treatment			Arable-Grassland				Arable-Heathland				Grassland-Heathland			
Response	d.f.	F	*p* value	d.f.	F	p value	B	d.f.	F	p value	B	d.f.	F	p value	B
*A. montana*	14, 163	3.21	**0.0002**	1,56	0.53	0.47	–	1,56	4.64	**0.036**	H > A	1,56	10.06	**0.0025**	H > G
C. rotundifolia*	14, 163	3.18	**0.0002**	1,56	0.20	0.65	–	1,56	3.11	0.083	(H > A)	1,56	1.72	0.20	–
*F. filiformis*	14, 163	1.99	**0.0214**	1,56	0.04	0.85	–	1,56	3.31	0.074	(H > A)	1,56	6.49	**0.014**	H > G
*C. capillaris*	14, 163	2.26	**0.008**	1,56	<0.01	0.95	–	1,56	5.16	**0.027**	H > A	1,56	2.77	0.10	–
*M. arvensis*	14, 163	18.09	**<0.0001**	1,56	9.54	**0.0031**	A > G	1,56	15.93	**0.0002**	A > H	1,56	32.63	**<0.0001**	G > H
*L. perenne*	14, 163	0.69	0.78	1,56	0.10	0.75	–	1,56	1.43	0.24	–	1,56	<0.01	0.99	–
Target biomass	14, 163	4.11	**<0.0001**	1,56	0.05	0.82	–	1,56	3.83	0.055	(H > A)	1,56	8.66	**0.0047**	H > G
Ruderal biomass	14, 163	2.78	**0.001**	1,56	8.52	**0.005**	A > G	1,56	0.21	0.65	–	1,56	8.05	**0.0063**	G > H
Total biomass	14, 163	2.78	**0.001**	1,56	8.73	**0.0046**	A > G	1,56	<0.01	0.98	–	1,56	4.45	**0.039**	G > H

*Log-transformed

**Fig. 3:**
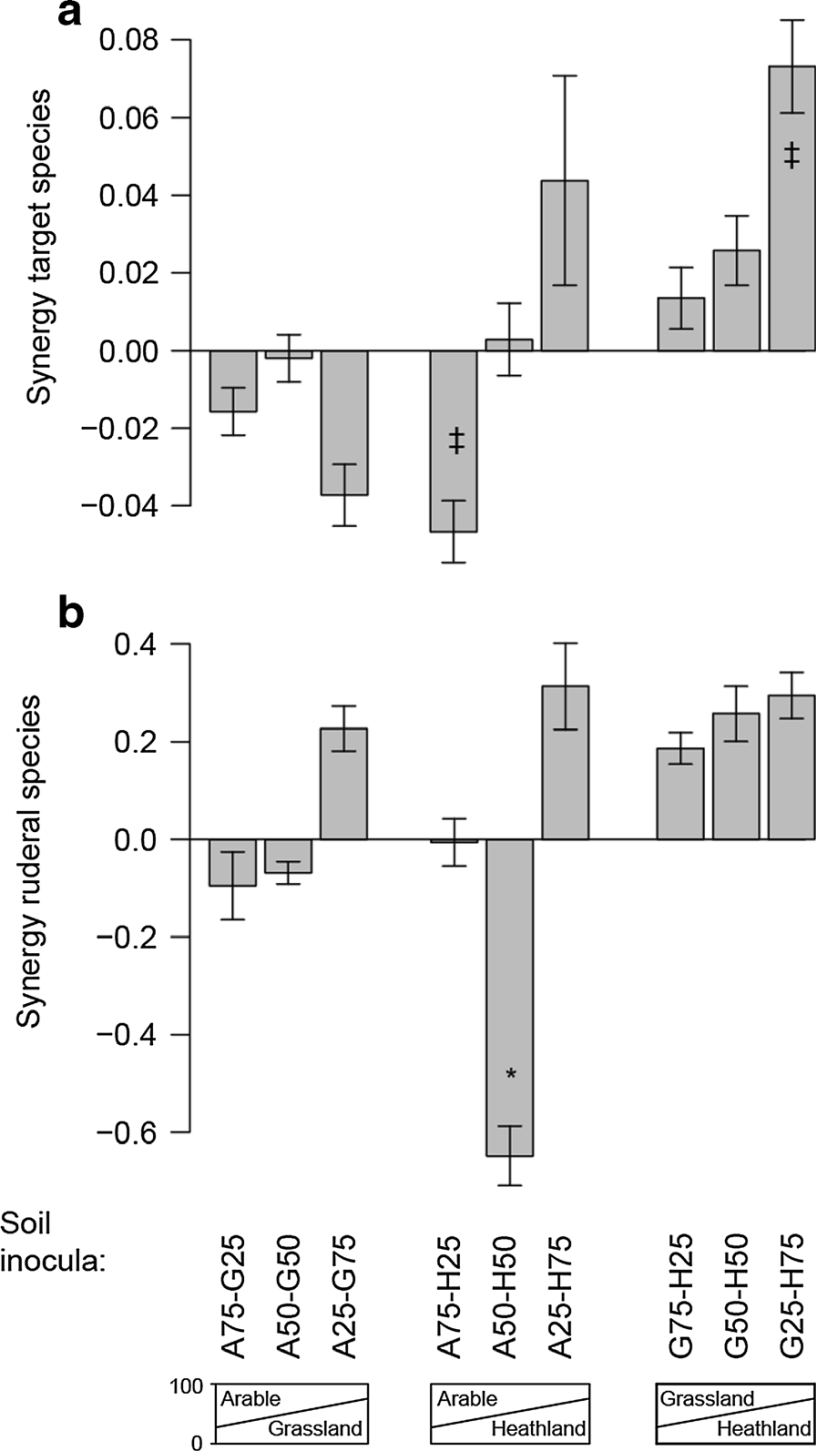
Synergetic effects of mixing inocula. Synergy is defined as the difference in (**a**) target or (**b**) ruderal biomass (g/pot; mean ± SE) observed in mixed inocula from that expected based on the pure inocula (i.e. dark bars in Fig. 2a, c). Asterisk indicates significant difference from zero at *p* < 0.05, ‡ same except *p* < 0.1

Mixing of inocula from different field types led to synergistic effects on the performance of plant groups (Fig. 3; Table S2). However, only the performance of ruderal species in the A50-H50 treatment was significantly lower than expected based on the pure inocula (Fig. 3). This response was driven by *M. arvensis*, while the other two ruderal species were not significantly affected (Fig. 2d). Due to the multiple testing correction this difference appears not significant in Fig. 2. However, when no such correction is applied, as is appropriate for planned contrasts in Fig. 3, ruderal biomass in A50-H50 is significantly lower than in all the other treatments in the replacement series. In addition, there was a trend (*p* < 0.1; Table S2) that G25-H75 and A75-H25 led to respectively higher and lower target species biomass than expected based on the pure inocula. There was no relationship between the synergistic effects of inocula on the ruderal and target species groups (Spearman’s rho = 0.078, *p* = 0.42).

Reponses to changes in inoculum composition were species specific (Fig. 2b, d; Table 1). The biomass of *M. arvensis* increased with higher amounts of arable soil and decreased with more heathland soil in the inoculum relative to grassland material (Table 1; Fig. 2d). Each of the three target species responded positively to a higher proportion of heathland material in the inoculum, but the highest biomass of *C. rotundifolia* was found in the A25-H75 treatment (Fig. 2b). More heathland material in the inoculum also led to higher *C. capillaris* biomass (Fig. 1d; Table 1). Only *L. perenne* was not significantly affected by changes in inoculum composition (Fig. 1d; Table 1).

## Discussion

We found that the composition of the soil inoculum affects the performance of both ruderal and late-successional target plant species. It is known that inoculation with late-successional soil enhances the performance of target plant species (De Deyn et al. 2003; Kardol et al. 2006; Carbajo et al. 2011; Middleton and Bever 2012). We now show that potential synergies among soil inocula exist when material from different successional stages is mixed. In particular, a 50:50 mixture of arable and heathland soil inoculum led to a lower performance of ruderal plant species than expected, which was driven by *M. arvensis* while the other two ruderal species did not respond significantly. In addition, two trends were observed: a 25:75 grassland-heathland mixture promoted target species biomass, while a 75:25 arable-heathland mixture repressed target species biomass relative to the performance expected based on the pure inocula. This shows that synergistic interactions among soil organisms upon inoculum source mixing can benefit restoration.

Importantly, there was no correlation between the synergistic effects of inoculum mixing on ruderal and target plants, neither positive nor negative. This suggests that synergistic effects depend on the soil inoculum source material and mixing ratio used. We found that the mixture of late-successional soil with arable soil most strongly repressed ruderal species growth. This was in line with our hypothesis that arable soil harbors high amounts of soil-borne enemies (Korthals et al. 2001; Verschoor et al. 2001; Holtkamp et al. 2008) to which early-successional plants are susceptible (Brown and Gange 1992; Fraser and Grime 1999). However, contrary to our hypothesis this mixture did not significantly improve the growth of target plants. The fact that synergistic effects were only detected in particular combinations of soils, as well as responding plant species, suggests that general rules based on successional changes may be insufficiently precise for broad application and that synergistic mixtures of inocula may need to be designed specifically for the area under restoration (Eviner and Hawkes 2008). Soils are inhabited by diverse assemblages of microbial and mesofaunal taxa (Bardgett and Van der Putten 2014), but they are also governed by strong competitive and trophic interactions (De Ruiter et al. 1995; Raaijmakers and Mazzola 2016) and soil communities are taxonomically highly distinct across ecosystems (Ettema and Wardle 2002; Bardgett and Van der Putten 2014). It is therefore likely that when these assemblages are suddenly brought into contact through mixing of inocula that new communities arise that are not simply the weighted sum of the communities in the original inocula (Rillig et al. 2016). Indeed, plant-soil community interactions have been reported to be non-additive when different soils are mixed (Hendriks et al. 2013; Wubs and Bezemer 2016; Ma et al. 2018).

Mid-succession grassland soil did not lead to intermediate performance of ruderal and target species, as their performance was mostly similar to that on full arable soil. Surprising is the observation that two independent implementations of the same treatment (G100), yielded differences in ruderal biomass. This suggests that samples taken from the same fields and thoroughly mixed may yield different soil communities when subsequently propagated in different experimental units, although this needs to be confirmed using molecular community analyses. Alternatively, intra-specific difference among plant individuals may have caused the difference in the two treatments, but we consider this to be unlikely given that individuals were randomly assigned to pots and treatments.

In our experiment plant species showed species-specific responses to the soil inocula. This was particularly true for the ruderal species, where *M. arvensis* responded negatively to heathland inocula, while *C. capillaris* was promoted by higher proportions of heathland inoculum, and *L. perenne* was unresponsive. The target species on the other hand all responded positively to higher proportions of heathland inoculum. This is in line with other inoculation studies (Carbajo et al. 2011; Middleton and Bever 2012) and may reflect their stronger association with soil-borne symbionts such as mycorrhizae (Reynolds et al. 2003). In the future, a wide range of plant species and soil inocula needs to be tested in combination to disentangle differential plant responses to changes in soil community composition and identify the soil taxa that drive these responses. This would allow screening for traits of plants and soil biota (see Eviner and Hawkes 2008), and their association with particular habitats, and allow predictions of optimized inocula composition for particular restoration goals.

While the target plants species had 33% more biomass when soils were inoculated with a high proportion of heathland material compared to no heathland material, they never became the dominant group in the plant community in this experiment (mean relative abundance varied between 5 and 10%). This can partly be explained by the slower growth rates typical of these species. In addition, in this study a relatively fertile, recently abandoned arable top-soil was used as a common background soil for the inoculations. It is well known that the outcome of plant-soil interactions depend on soil fertility (Bezemer et al. 2006; Eviner and Hawkes 2008; Van der Putten et al. 2016). For instance, soil biota tend to have smaller effects on plant performance in more fertile soils (De Deyn et al. 2004; Carbajo et al. 2011; Wubs et al. 2016; Wubs and Bezemer 2018). Under high nutrient conditions plants may be less depended on for instance mycorrhizae and better able to defend against antagonists (De Deyn et al. 2004; Van der Bij et al. 2016), suggesting that inoculation effects may be more pronounced in infertile soils. In general, there is a need to screen for the effectiveness of soil inoculations across environmental gradients to evaluate their potential (Eviner and Hawkes 2008).

Plant-soil interactions can be mediated by both abiotic and biotic factors (Ehrenfeld et al. 2005). We think that the effect of differences in abiotic conditions among the pots treated with the different inocula was of negligible importance for two reasons. First, due to the dilution of the inocula in the common, relatively fertile, background soil there was limited scope for nutrient limitation. Secondly, the proportion of heathland material in the mixture contributed most to target species plant performance. However, the heathlands had the lowest concentration of nutrients and the lowest acidity among the sampled inocula. Therefore, if abiotic differences were the sole driving factor we would have expected plant performance to be lowest on 100% heathland inoculum, which was not observed. In a comparable inoculation study from the same study region both soil abiotic and biotic variables were measured and they showed that plant responses correlated most strongly with biotic drivers, mainly fungal and bacterivorous nematode abundance (Carbajo et al. 2011). Therefore, we propose that the observed differences in plant performance were mainly due to the inoculated soil communities, although we cannot rule out abiotic effects.

We conclude that soil inocula differentially affect the performance of ruderal and late-successional target plant species, also under high soil fertility. Target plant species benefited from inoculation with heathland material, while responses of ruderal species were variable. Mixing inocula from different ecosystems led to synergistic effects for restoration, but this was highly depended on the particular combination of inocula applied. As a next step, a broad screen of mixed and unmixed soil inocula needs to be tested across environmental gradients to generally assess the effectiveness of soil inoculation for nature restoration.

## Electronic supplementary material

ESM 1 (DOCX 103 kb)

## References

[CR1] Adbi H, Williams LJ, Salkind N (2010). Contrast analysis. Encyclopedia of research design. Sage.

[CR2] Bardgett RD, Van der Putten WH (2014). Belowground biodiversity and ecosystem functioning. Nature.

[CR3] Barni E, Siniscalco C (2000). Vegetation dynamics and arbuscular mycorrhiza in old-field successions of the western Italian Alps. Mycorrhiza.

[CR4] Bauer JT, Mack KML, Bever JD (2015). Plant-soil feedbacks as drivers of succession: evidence from remnant and restored tallgrass prairies. Ecosphere.

[CR5] Benjamini Y, Hochberg Y (1995). Controlling the false discovery rate: a practical and powerful approach to multiple testing. Journal of the Royal Statistical Society B.

[CR6] Bezemer TM, Lawson CS, Hedlund K (2006). Plant species and functional group effects on abiotic and microbial soil properties and plant-soil feedback responses in two grasslands. J Ecol.

[CR7] Brown VK, Gange AC (1992). Secondary plant succession: how is it modified by insect herbivory?. Vegetatio.

[CR8] Carbajo V, den Braber B, van der Putten WH, De Deyn GB (2011). Enhancement of late successional plants on ex-arable land by soil inoculations. PLoS One.

[CR9] Castle SC, Lekberg Y, Affleck D, Cleveland CC (2016). Soil abiotic and biotic controls on plant performance during primary succession in a glacial landscape. J Ecol.

[CR10] De Deyn GB, Raaijmakers CE, Zoomer HR (2003). Soil invertebrate fauna enhances grassland succession and diversity. Nature.

[CR11] De Deyn GB, Raaijmakers CE, Van der Putten WH (2004). Plant community development is affected by nutrients and soil biota. J Ecol.

[CR12] De Ruiter PC, Neutel A-M, Moore JC (1995). Energetics, patterns of interaction strengths, and stability in real ecosystems. Science.

[CR13] De Wit CT (1960). On competition. Verslag Landbouwkundige Onderzoekingen.

[CR14] Ehrenfeld JG, Ravit B, Elgersma K (2005). Feedback in the plant-soil system. Annu Rev Environ Resour.

[CR15] Ettema CH, Wardle DA (2002). Spatial soil ecology. Trends Ecol Evol.

[CR16] Eviner VT, Hawkes CV (2008). Embracing variability in the application of plant–soil interactions to the restoration of communities and ecosystems. Restor Ecol.

[CR17] Fraser LH, Grime JP (1999). Interacting effects of herbivory and fertility on a synthesized plant community. J Ecol.

[CR18] Frouz J, Toyota A, Mudrák O, Jílková V, Filipová A, Cajthaml T (2016). Effects of soil substrate quality, microbial diversity and community composition on the plant community during primary succession. Soil Biol Biochem.

[CR19] Hannula SE, Morriën E, de Hollander M, van der Putten WH, van Veen JA, de Boer W (2017). Shifts in rhizosphere fungal community during secondary succession following abandonment from agriculture. ISME J.

[CR20] Harris J (2009). Soil microbial communities and restoration ecology: facilitators or followers?. Science.

[CR21] Helgason T, Daniell TJ, Husband R, Fitter AH, Young JPW (1998). Ploughing up the wood-wide web?. Nature.

[CR22] Hendriks M, Mommer L, De Caluwe H (2013). Independent variations of plant and soil mixtures reveal soil feedback effects on plant community overyielding. J Ecol.

[CR23] Hobbs RJ, Harris JA (2001). Restoration ecology: repairing the Earth’s ecosystems in the new millennium. Restor Ecol.

[CR24] Holtkamp R, Kardol P, van der Wal A, Dekker SC, van der Putten WH, de Ruiter PC (2008). Soil food web structure during ecosystem development after land abandonment. Appl Soil Ecol.

[CR25] Janos DP (1980). Mycorrhizae influence tropical succession. Biotropica.

[CR26] Johnson NC, Zak DR, Tilman D, Pfleger FL (1991). Dynamics of vesicular-arbuscular mycorrhizae during old field succession. Oecologia.

[CR27] Kardol P, Wardle DA (2010). How understanding aboveground-belowground linkages can assist restoration ecology. Trends Ecol Evol.

[CR28] Kardol P, Bezemer TM, van der Wal A, van der Putten WH (2005). Successional trajectories of soil nematode and plant communities in a chronosequence of ex-arable lands. Biol Conserv.

[CR29] Kardol P, Bezemer TM, Van der Putten WH (2006). Temporal variation in plant-soil feedback controls succession. Ecol Lett.

[CR30] Kardol P, Cornips NJ, van Kempen MML, Bakx-Schotman JMT, van der Putten WH (2007). Microbe-mediated plant-soil feedback causes historical contingency effects in plant community assembly. Ecol Monogr.

[CR31] Kardol P, De Deyn GB, Laliberté E (2013). Biotic plant–soil feedbacks across temporal scales. J Ecol.

[CR32] Korthals GW, Smilauer P, Van Dijk C, Van der Putten WH (2001). Linking above- and below-ground biodiversity: abundance and trophic complexity in soil as a response to experimental plant communities on abandoned arable land. Funct Ecol.

[CR33] Lenth R (2015) lsmeans: Least-Squares Means. R package version 2:20–23. https://CRAN.R-project.org/package=lsmeanshttps://CRAN.R-project.org/package=lsmeans

[CR34] Ma Hai-kun, Pineda Ana, van der Wurff Andre W. G., Bezemer T. Martijn (2018). Synergistic and antagonistic effects of mixing monospecific soils on plant-soil feedbacks. Plant and Soil.

[CR35] Middleton EL, Bever JD (2012). Inoculation with a native soil community advances succession in a grassland restoration. Restor Ecol.

[CR36] Middleton EL, Richardson S, Koziol L, Palmer CE, Yermakov Z, Henning JA, Schultz PA, Bever JD (2015). Locally adapted arbuscular mycorrhizal fungi improve vigor and resistance to herbivory of native prairie plant species. Ecosphere.

[CR37] Morriën E, Hannula SE, Snoek LB, Helmsing NR, Zweers H, de Hollander M, Soto RL, Bouffaud ML, Buée M, Dimmers W, Duyts H, Geisen S, Girlanda M, Griffiths RI, Jørgensen HB, Jensen J, Plassart P, Redecker D, Schmelz RM, Schmidt O, Thomson BC, Tisserant E, Uroz S, Winding A, Bailey MJ, Bonkowski M, Faber JH, Martin F, Lemanceau P, de Boer W, van Veen JA, van der Putten WH (2017). Soil networks become more connected and take up more carbon as nature restoration progresses. Nat Commun.

[CR38] Oksanen J, Blanchet FG, Kindt R (2018). Vegan: community ecology package. R package version.

[CR39] Pinheiro JC, Bates DM (2000). Mixed-effects models in S and S-PLUS.

[CR40] Pinheiro JC, Bates DM, DebRoy S et al (2017) nlme: Linear and Nonlinear Mixed Effects Models. http://CRAN.R-project.org/package=nlme. Accessed 27 Feb 2017

[CR41] R Core Team (2017). R: a language and environment for statistical computing.

[CR42] Raaijmakers JM, Mazzola M (2016). Soil immune responses. Science.

[CR43] Rasmann S, Bauerle TL, Poveda K, Vannette R (2011). Predicting root defence against herbivores during succession. Funct Ecol.

[CR44] Reynolds HL, Packer A, Bever JD, Clay K (2003). Grassroots ecology: plant–microbe–soil interactions as drivers of plant community structure and dynamics. Ecology.

[CR45] Rillig MC, Lehmann A, Aguilar-Trigueros CA, Antonovics J, Caruso T, Hempel S, Lehmann J, Valyi K, Verbruggen E, Veresoglou SD, Powell JR (2016). Soil microbes and community coalescence. Pedobiologia.

[CR46] Schaminée JHJ, Stortelder AH., Weeda EJ (1996) De vegetatie van Nederland. Deel 3. Plantengemeenschappen van graslanden, zomen en droge heiden. Opulus Press, Uppsala

[CR47] Schaminée JHJ, Weeda EJ, Westhoff V (1998) De vegetatie van Nederland. Deel 4. Plantengemeenschappen van de kust en van binnenlandse pioniersmilieus. Opulus Press, Uppsala

[CR48] Tsiafouli MA, Thébault E, Sgardelis SP, de Ruiter PC, van der Putten WH, Birkhofer K, Hemerik L, de Vries FT, Bardgett RD, Brady MV, Bjornlund L, Jørgensen HB, Christensen S, Hertefeldt TD’, Hotes S, Gera Hol WH, Frouz J, Liiri M, Mortimer SR, Setälä H, Tzanopoulos J, Uteseny K, Pižl V, Stary J, Wolters V, Hedlund K (2015). Intensive agriculture reduces soil biodiversity across Europe. Glob Chang Biol.

[CR49] Van der Bij AU, Pawlett M, Harris JA (2016). Soil microbial community assembly precedes vegetation development after drastic techniques to mitigate effects of nitrogen deposition. Biol Conserv.

[CR50] Van der Putten WH, Bradford MA, Brinkman EP (2016). Where, when and how plant-soil feedback matters in a changing world. Funct Ecol.

[CR51] Van der Wal A, Van Veen JA, Smant W (2006). Fungal biomass development in a chronosequence of land abandonment. Soil Biol Biochem.

[CR52] Verschoor B.C, de Goede R.G.M, de Vries F.W, Brussaard L (2001). Changes in the composition of the plant-feeding nematode community in grasslands after cessation of fertiliser application. Applied Soil Ecology.

[CR53] Vitousek PM, Mooney HA, Lubchenco J, Melillo JM (1997). Human domination of Earth’s ecosystems. Science.

[CR54] Weigelt A, Jolliffe P (2003). Indices of plant competition. J Ecol.

[CR55] Wubs ERJ, Bezemer TM (2016). Effects of spatial plant–soil feedback heterogeneity on plant performance in monocultures. J Ecol.

[CR56] Wubs ERJ, Bezemer TM (2018). Temporal carry-over effects in sequential plant–soil feedbacks. Oikos.

[CR57] Wubs ERJ, Van der Putten WH, Bosch M, Bezemer TM (2016). Soil inoculation steers restoration of terrestrial ecosystems. Nature Plants.

[CR58] Zuur AF, Ieno EN, Walker NJ (2009). Mixed effects models and extensions in ecology with R. springer science+business media. LLC.

