# Supplementary material for: Potential for synergy in soil inoculation for nature restoration by mixing inocula from different successional stages
Source: Plant Soil. 2018 Oct 3;433(1):147–56. doi: 10.1007/s11104-018-3825-0 (PMC6405189; doi:10.1007/s11104-018-3825-0)
Supplement: Supplementary file 1 — (DOCX 103 kb) [file 11104_2018_3825_MOESM1_ESM.docx]

**Supplementary information**

Table S1. Background information on the location and age of the sampled fields, including abiotic conditions (mean±SE, n = 3 in all cases).

| Type of field | Field triplet | Field name | Latitude (°N) | Longitude (°E) | Abandoned (Year) | Successional age in 2015 (Years) | Organic matter  content  (% w:w) | Acidity  (pH in H_2_O) | PO_4_-P  (mg/kg) | NO_3_-N  (mg/kg) | NH_4_–N  (mg/kg) |
| --- | --- | --- | --- | --- | --- | --- | --- | --- | --- | --- | --- |
| Recipient |  |  |  |  |  |  |  |  |  |  |  |
| Ex-arable field | - | Reijerscamp | 52.015 | 5.777 | 2006 | 9 | 5.92±0.21 | 5.87±0.14 | 78.32±6.71 | 0.71±0.37 | 7.23±0.64 |
| Inoculum donors | |  |  |  |  |  |  |  |  |  |  |
| Arable field | A | Reemsterakker | 52.046 | 5.807 | - | 0 | 3.20±0.10 | 5.36±0.14 | 58.44±2.25 | 6.27±2.00 | 2.40±0.99 |
|  | B | Reijerscamp akker | 52.017 | 5.790 | - | 0 | 5.23±0.11 | 5.48±0.07 | 53.41±2.54 | 4.24±0.95 | 2.18±0.21 |
|  | C | Sinderhoeve | 51.998 | 5.752 | - | 0 | 5.69±0.30 | 4.05±0.01 | 120.27±13.43 | 6.80±0.29 | 2.37±0.08 |
| Grassland | A | Dennekamp | 52.029 | 5.802 | 1982 | 33 | 4.66±0.08 | 4.72±0.04 | 47.19±2.28 | 3.88±0.53 | 2.87±0.41 |
|  | B | Mosschelse Veld West | 52.073 | 5.735 | 1985 | 30 | 3.37±0.02 | 4.59±0.01 | 52.42±1.08 | 5.72±0.52 | 5.14±2.00 |
|  | C | Wolfhezer Veld | 51.995 | 5.791 | 1988 | 27 | 5.07±0.26 | 4.28±0.02 | 39.14±3.10 | 7.30±1.38 | 1.96±0.34 |
| Heathland | A | Reemsterheide | 52.041 | 5.802 | - | >200 | 8.10±0.17 | 3.11±0.02 | 2.16±0.48 | 0.51±0.04 | 4.90±0.10 |
|  | B | Mosschelse Veld East | 52.069 | 5.744 | - | >200 | 6.84±1.44 | 3.60±0.02 | 2.73±1.20 | 0.45±0.15 | 3.63±0.19 |
|  | C | Doorwerthse Heide | 51.992 | 5.775 | - | >200 | 7.73±0.29 | 3.66±0.01 | 1.02±0.14 | 1.23±0.09 | 6.11±0.49 |


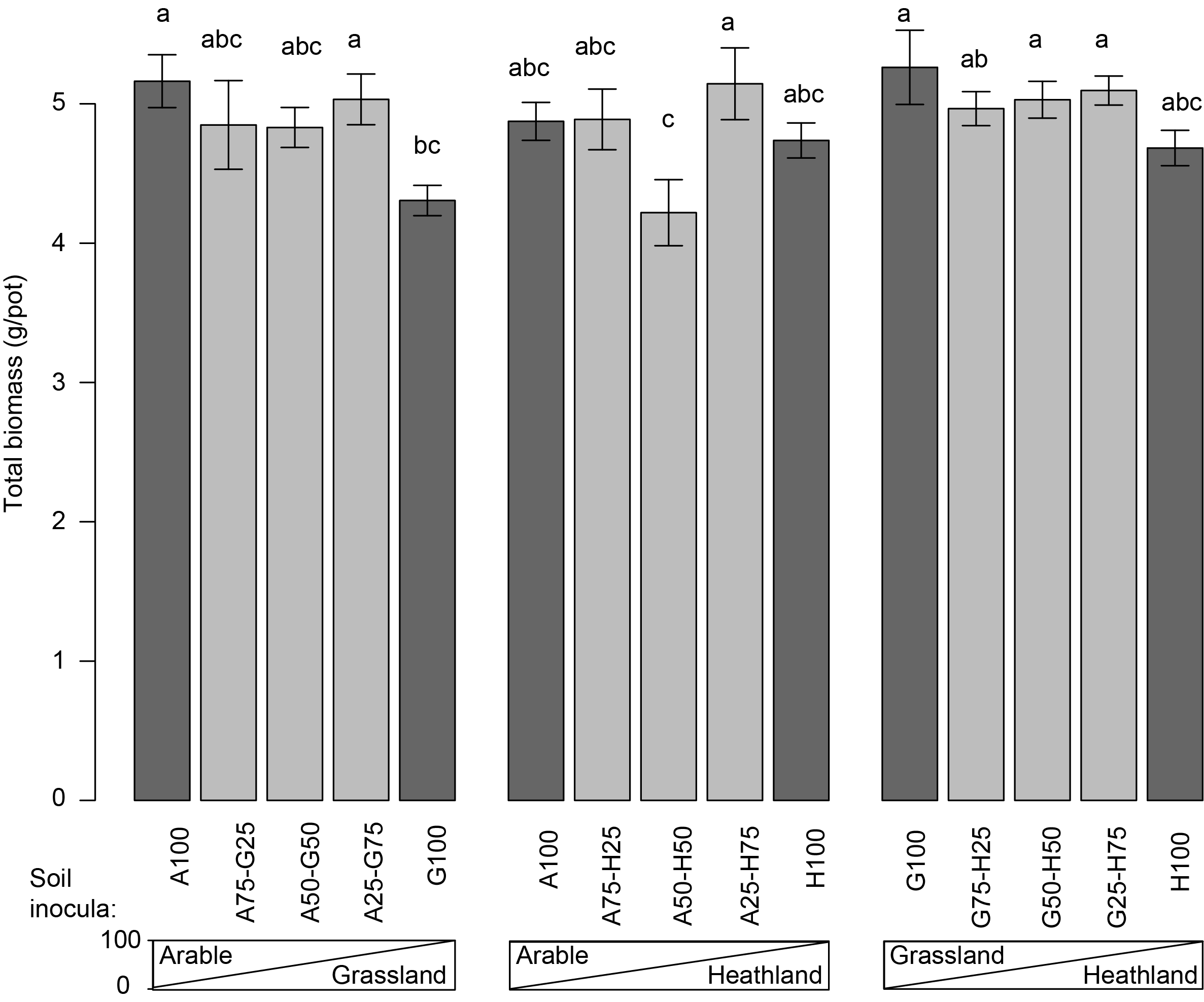


Fig. S1. Total shoot biomass (mean±SE) in response to each of the inoculum treatments. Different letters indicate significant differences among the treatments.

Table S2. Results of the planned contrast analyses for synergistic effects of soil inocula mixing on the performance of target and ruderal plant species. Planned contrasts were tested within LMMs to maximise statistical power.

|  | Target spp. | | Ruderal spp. | |
| --- | --- | --- | --- | --- |
| Contrast | t_97_ | p-value | t_97_ | p-value |
| A075G025 vs. Pure inoc. | -0.74 | 0.46 | -0.34 | 0.74 |
| A050G050 vs. Pure inoc. | -0.09 | 0.93 | 0.45 | 0.65 |
| A025G075 vs. Pure inoc. | -1.36 | 0.18 | 1.29 | 0.20 |
| A075H025 vs. Pure inoc. | -1.70 | *0.0923* | -0.03 | 0.98 |
| A050H050 vs. Pure inoc. | 0.09 | 0.93 | -2.79 | **0.0063** |
| A025H075 vs. Pure inoc. | 0.47 | 0.64 | 1.02 | 0.31 |
| G075H025 vs. Pure inoc. | 0.49 | 0.62 | 1.17 | 0.25 |
| G050H050 vs. Pure inoc. | 0.83 | 0.41 | 1.23 | 0.22 |
| G025H075 vs. Pure inoc. | 1.77 | *0.0802* | 1.64 | 0.10 |
